# Structural and viscosity studies of dendritic hyper branched polymer as viscosity index improvers

**DOI:** 10.1186/s13065-024-01206-2

**Published:** 2024-05-30

**Authors:** Reham I. El-shazly, Rasha S. Kamal, Reem K. Farag

**Affiliations:** Department of Petroleum Applications, Egyptian Petroleum Research Institute, Nasr City, Cairo Egypt

**Keywords:** Hyperbranched polymer, DLS, Viscosity index improver, Michael addition

## Abstract

Star-like structural compounds were synthesized from different moles % of either dodecyl acrylate or triethylenetetramine using a one-pot commercial synthesis technique. The polymers that were created had various terminations. Fourier Transform Infrared (FTIR) spectroscopy and ^1^HNMR were used to verify the produced polymers' chemical composition with different terminations. Furthermore, by analysis of Dynamic Light Scattering (DLS), the size and distribution of the synthesised branched polymers were evaluated. Using a Gel-permeation chromatograph, the modified hyperbranched polymer's molecular weight, synthesized with various end points, were assessed. The unorganized structured prepared compounds with various molar feed ratios dodecyl acrylate: triethylenetetramine (DDA: TETA) was designed as A, B, C, D and E. Moreover, the synthesized additives function as viscosity index improvers (VII). As the concentration of polymeric additives increases, it leads to higher VI values. Similarly, with the increase in percentage of triethylenetetramine in the prepared hyperbranched polymers, the VI also increases. Notably, the most effective VI achieved is (E) = 212. It is noteworthy that all the synthesized hyperbranched polymers exhibited Newtonian behavior in the rheological study.

## Introduction

When two objects are moving relative to one another, a substance is used to ensure as much smooth functioning as possible, which refers to lubrication. According to factors such as operating speed, temperature, ambient conditions, and others, the quantities and types of lubricants for rolling bearings are chosen. Lubricants must be routinely replaced or oiled because they have reached the end of their service lifetime or have become contaminated with undesirable substances and are no longer capable of performing their intended function. In order to prevent direct contact between the material surfaces, the lubricant film develops a thick coating between moving or sliding surfaces and fills in any imperfections on those surfaces. Friction is subsequently decreased. Under operating conditions, the lubricant should have the least possible viscosity (to decrease internal resistance between the lubricant particles), while remaining in place and separating the surfaces. Regular hydrocarbon lubricants are combined with certain long chain polymers to preserve the viscosity of the oil throughout the year [1-3]. The term "viscosity" refers to a liquid's resistance to flow. It is the most significant attribute of any lubricating oil since it is the primary predictor of the lubricant's operating qualities. A liquid oil coating cannot be maintained between two moving or sliding surfaces if the viscosity of the oil is too low. On the other side, if the oil's viscosity is excessively high, there will be an excessive amount of friction. Viscosity is impacted by temperature: Since the viscosity of liquids reduces as temperature rises, the lubricating oil thins out as operational temperature rises. As a result, excellent lubricating oil should have a viscosity that does not significantly alter with temperature change, so that it may be used continually in a variety of temperature-related situations [4-7]. An arbitrary scale called the viscosity index is used to gauge how quickly lubricating oil viscosity varies with temperature (V. I). It has a low viscosity index if the viscosity of lubricating oil rapidly decreases when the temperature is raised. On the other hand, lubricating oil has a high viscosity index if temperature increases only marginally, impacts its viscosity [8-11]. Hyperbranched polymers are one-of-a-kind high branching density polymers, a significant quantity of terminal reactive units and nano-sized repeated branched building blocks. These significant properties opened the door to a brand-new class of materials with uses in physics, biotechnology, and life science [12-17]. Hyperbranched polymers have been widely used in the petroleum industry as demulsifiers [18], corrosion inhibitors, scale inhibitors [19]. , pipeline coating [20], oil spill dispersants [21], and asphaltene dispersants [22, 23]. Our previous work is [5-7, 24-26] In this study, hyperbranched polymer was created using a simple and widely commercialized method of Michale addition and amidation repeated. There was a change made to enhance the synthesis's reliability. The present hyperbranched polymers have been successfully synthesized via different molar ratios of dodecyl acrylate and triethylenetetramine in nanoscale and additionally characterized. The synthesized branched polymers have been used in three terminations with different terminations, and assessed as viscosity index improver.

## Experimental

### Chemicals

Lube oil (SAE 30) is a free additive base oil obtained from the Petroleum Co-operative Society. Dodecyl acrylate (DDA), triethylenetetramine (TETA), formaldehyde (37%) and formic acid (88%), which were used without further purification are provided by Sigma Aldrich. Unless otherwise specified, the other chemicals were all reagent grade.

### Synthesis of hyperbranched poly (amido amine)

A solution of different moles% triethylenetetramine is dissolved in 25 ml of acetone in a one-neck flask. Then, different moles% of a methyl acrylate dodecyl acrylate (DDA) were dropwise added into the reaction mixture for 5 h at 0 °C in a nitrogen atmosphere. After being stirred for 30 min at the same temperature, the mixture was left to warm up for 48 h at room temperature. In vacuum, at room temperature, the rotary evaporator evaporates the methanol solvent, leaving behind a sticky yellow substance. This yellow product was cleaned three times with diethyl ether and stored overnight in a container that was firmly closed [27] to give four samples of branched polymers as represented in Table (1).

**Table 1:** Abbreviation of the prepared compounds and the different ratio of the reactants

Abbreviation	Experiment	Dodecyl acrylate ratio	Triethylenetetramine ratio
**A**	dodecyl acrylate + riethylenetetramine	1	1
**B**	dodecyl acrylate + triethylenetetramine	2	1
**C**	dodecyl acrylate + triethylenetetramine	3	1
**D**	dodecyl acrylate + triethylenetetramine	1	2
**E**	dodecyl acrylate + triethylenetetramine	1	3

### H-PAMAM tertiary amine synthesis

Because the polymer has the ability to absorb into the column, Gel Permeation Chromatography (GPC) does not directly measure the molecular weights of the five hyperbranched samples [28]. The solution was accomplished by switching the primary amine in the terminal groups to tertiary amine, as previously explained by Liang and colleagues [27]. . 10 g of cold formic acid (88%), followed by 10 g of formaldehyde (37%), received a progressive addition of 0.5 g of H-PAMAM solution. A rotary evaporator was used to extract the solvent under vacuum after around nine hours of refluxing at 90-100 °C, and the resulting slurry was then dried at 40 °C while under vacuum.

### Preparation of the hyperbranched polymer

Preparation of the reactants (dodecyl acrylate and triethylenetetramine) in different ratio as shown in Table (1) were stirred for 8 h in an ice bath using 5 ml acetone as solvent.

### Characterization of the synthesized hyperbranched polymer

#### FTIR analysis

An FTIR Nicolet iS10 spectrometer was used to confirm the chemical structures of the produced samples (Thermo Fisher Scientific, USA). Before forming an analytical disc, the samples were thoroughly blended with potassium bromide (KBr). Model Top 961 of the Mattson Infinity Series, a kind of spectrometer used by the Egyptian Petroleum Research Institute (EPRI).

#### Spectroscopic ^1^HNMR analysis

The ^1^HNMR spectra of the synthesized compounds were measured using the following equipment: a 400-Megahertz magnet, a Varian model mercury plus spectrometer, and a Varian 5-millimeter probe.

#### Dynamic light scattering (DLS)

Dynamic Light Scattering (DLS) at 25 °C and a Zeta Sizer Nano from ZS-Malvern Corporation, UK, were used at the Egyptian Petroleum Research Institute (EPRI) to measure the particle size and size distribution of nano-clay-based materials.

#### Differential scanning calorimetry (DSC)

The structural changes of the prepared samples with thermal treatment were studied using differential scanning calorimetry (DSC). DSC-60 detector instrument heated 1.610 mg of sample to 400 °C at a rate of 10 °C min^− 1^ with an aluminium valve cell and a N_2_ atmosphere.

### Evaluation as viscosity index improver

Utilizing the calculation method described in ASTMD 2270-87, the viscosity-temperature behavior of polymer nanocomposite was characterized. Kinematic viscosity at 40 °C and 100 °C are used to determine the viscosity index, a widely accepted and utilized measure of the fluctuation in kinematic viscosity caused by differences in a petroleum product's temperature between 40 °C and 100 °C.

### Study rheological properties

At the Egyptian Petroleum Research Institute, we use the Modular Compact Rheometer Model Type 502 (Anton Paar) to study rheological properties.

## Results and discussion

**Fig. 1:**
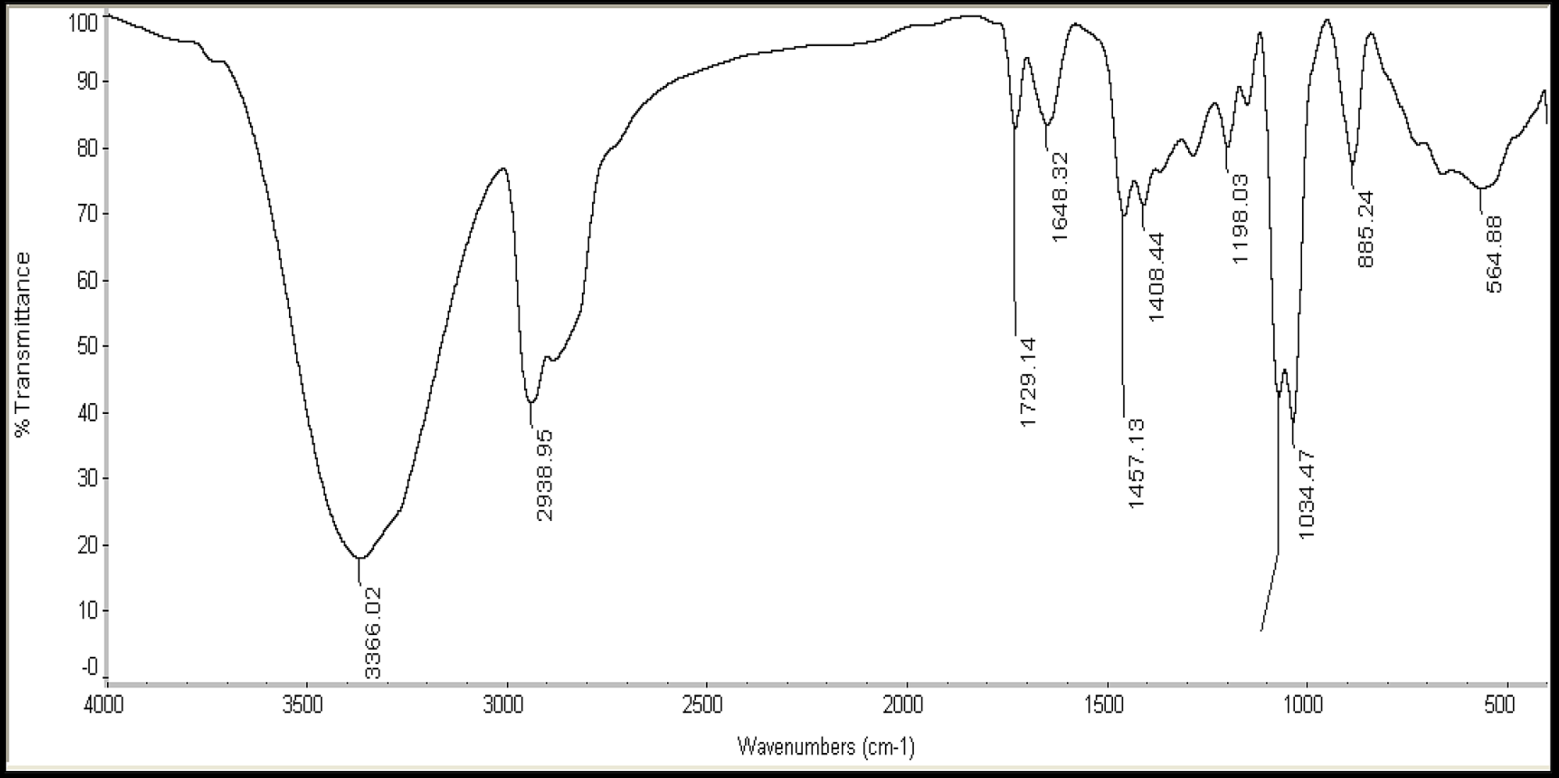
I.R. Spectrum of Chemical structure of hyper branched prepared compound A

The preparation of hyperbranched polymers pursued a technique mentioned by Gao [21]. With the diagram of the optimal two-step reaction in Fig. 1. In current research, a modification was developed to enhance the synthesis's reliability. First, at room temperature, Michael addition of DA to TETA yields intermediates 1 or 2. Although amidation reaction is unavoidable between (DA: TETA), at lower temperatures, Michael in addition tends to react faster. Gradually increasing the temperature causes intermediates 1 or 2 to form an amine terminated hyperbranched polymer, as shown in Table (1). To isolate the methanol, the reaction took place in a rotating evaporator under vacuum and at high temperature, amidation products, and solvent as quickly as possible.

**Scheme 1:**
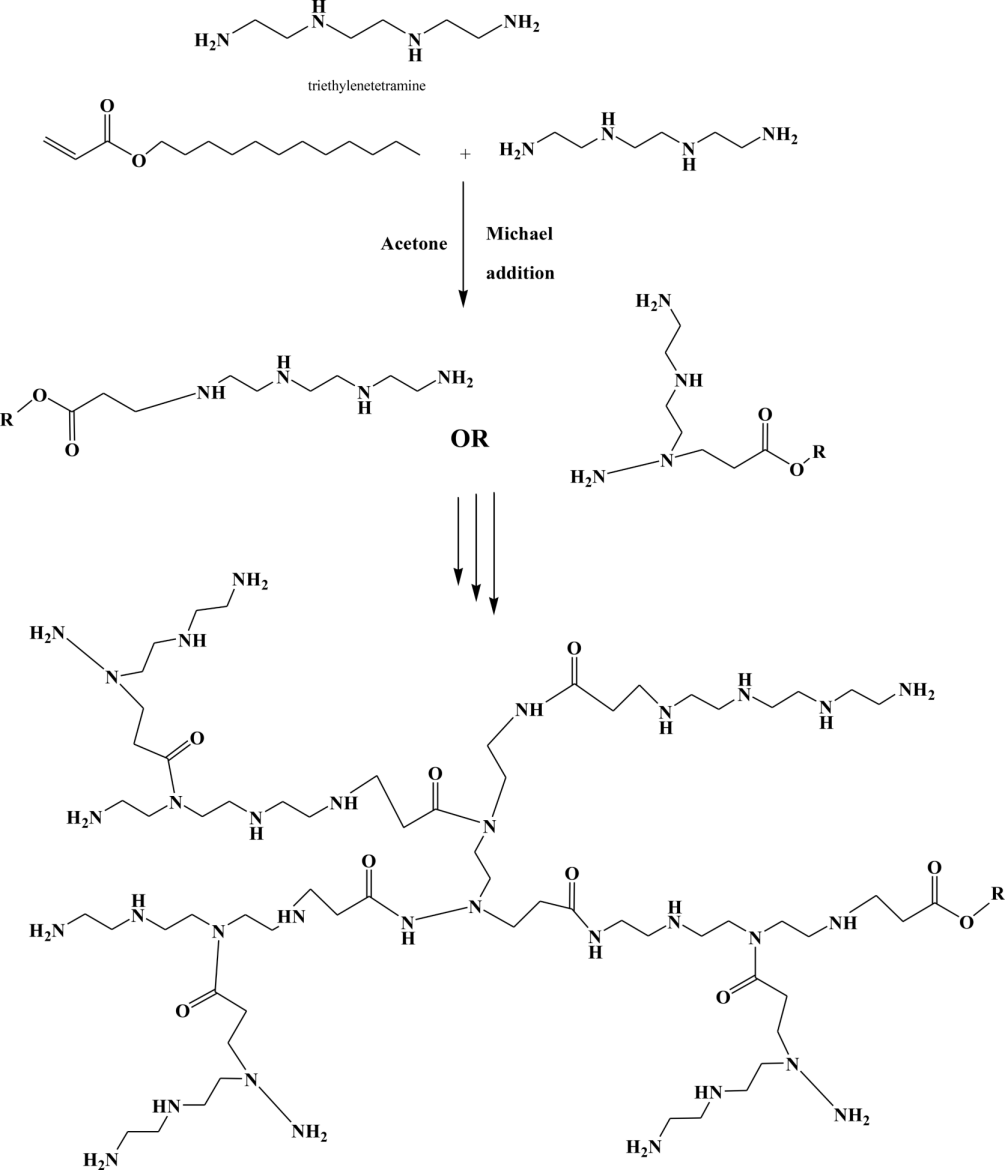
Preparation of hyperbranched polymer

When aiming to limit the molecular weight distribution, diethyl ether purification can effectively eliminate any remaining monomers or low molecular weight products. The reaction scheme is like Scheme (1). FTIR is an effective instrument for analysing how prepared hyperbranched polymers behave.

**Fig. 2:**
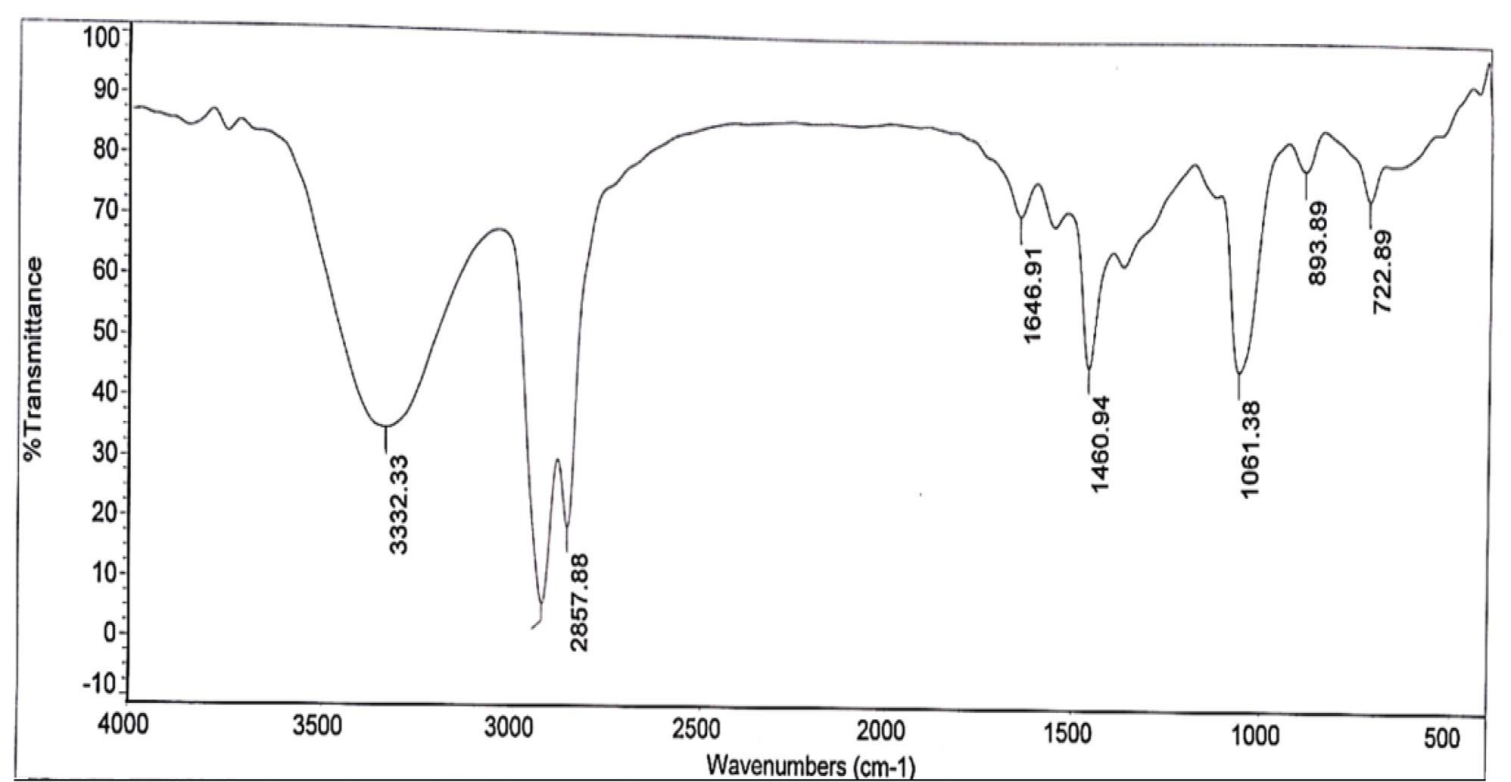
I.R. Spectrum of Chemical structure of hyper branched prepared compound E

As shown in Figs. (1-2), the **FTIR spectra** of the prepared samples have two reaction modes, with the disappearance of the peak at 1730 cm^− 1^ was attributed to C = O of DA. Meanwhile, -CONH has peaks at 1650 cm^− 1^ and 1558 cm^− 1^, which were previously assigned to -CONH ]18] These two patterns demonstrated how intermediates 1 and 2 came together over time to create the final A or E samples. Methyl acrylate might be reacted with a substance having a hydroxyl group and one main amino or two secondary amino groups to produce a methoxycarbonyl-terminated hyperbranched polymer. The feed ratios clearly affect the polymerization process. In this study, the E sample was synthesised using a method described by [21], Table 1 shows the ratio of mole feed of dodecyl acrylate to TETA monomer. The FTIR spectrum of the E sample revealed a broad band for NH groups at 3200-3600 cm^− 1^, a strong band for CH alkanes at 3000 -2850 cm^− 1^, a C-N band at 1350 -1000 cm^− 1^, an O = C-N band of amide at 1648 cm^− 1^ and a C-O band at 1250 -1000 cm^− 1^.

**Fig. 3:**
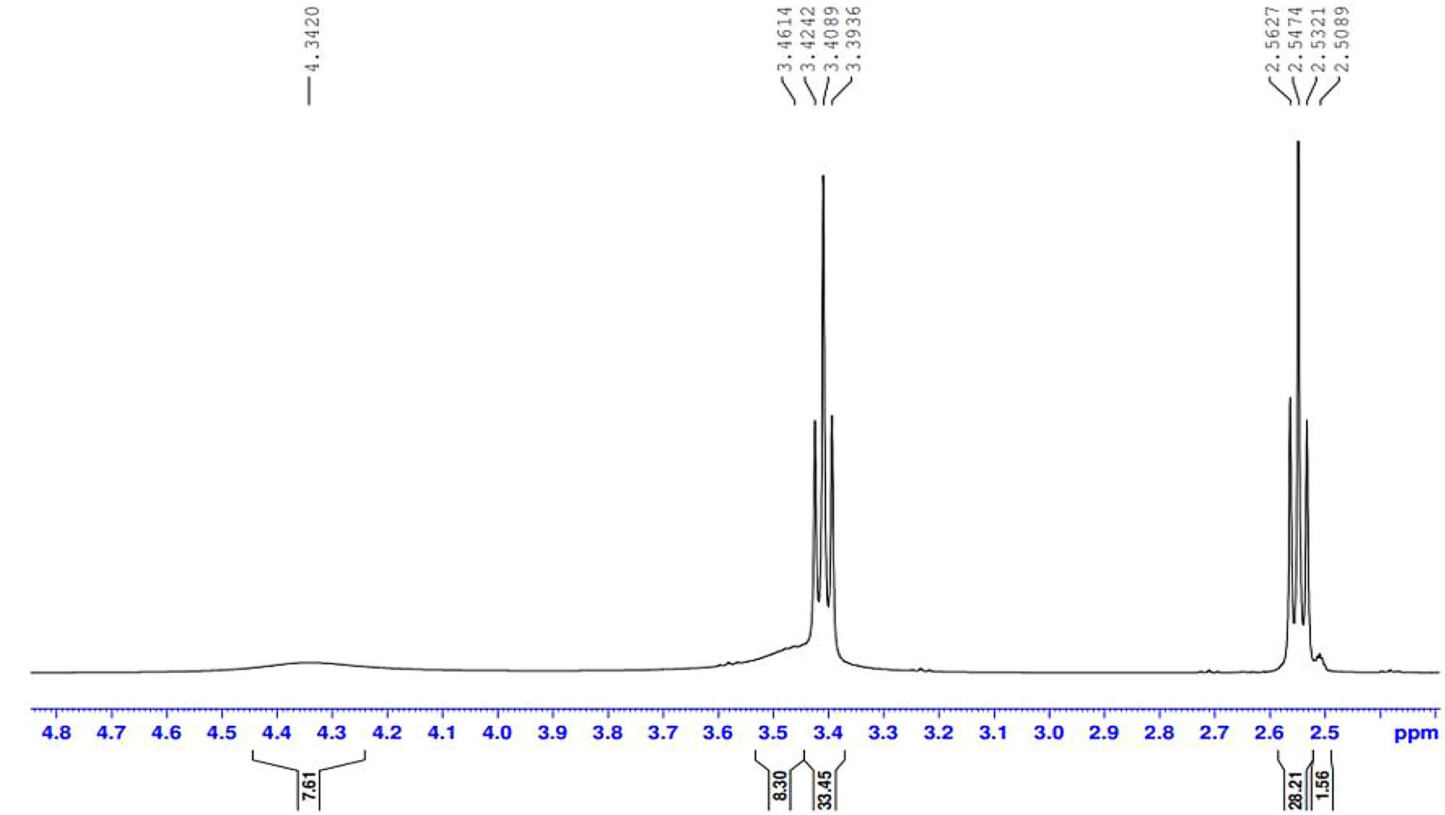
^1^HNMR spectrum of the prepared compound (A)

**Fig. 4:**
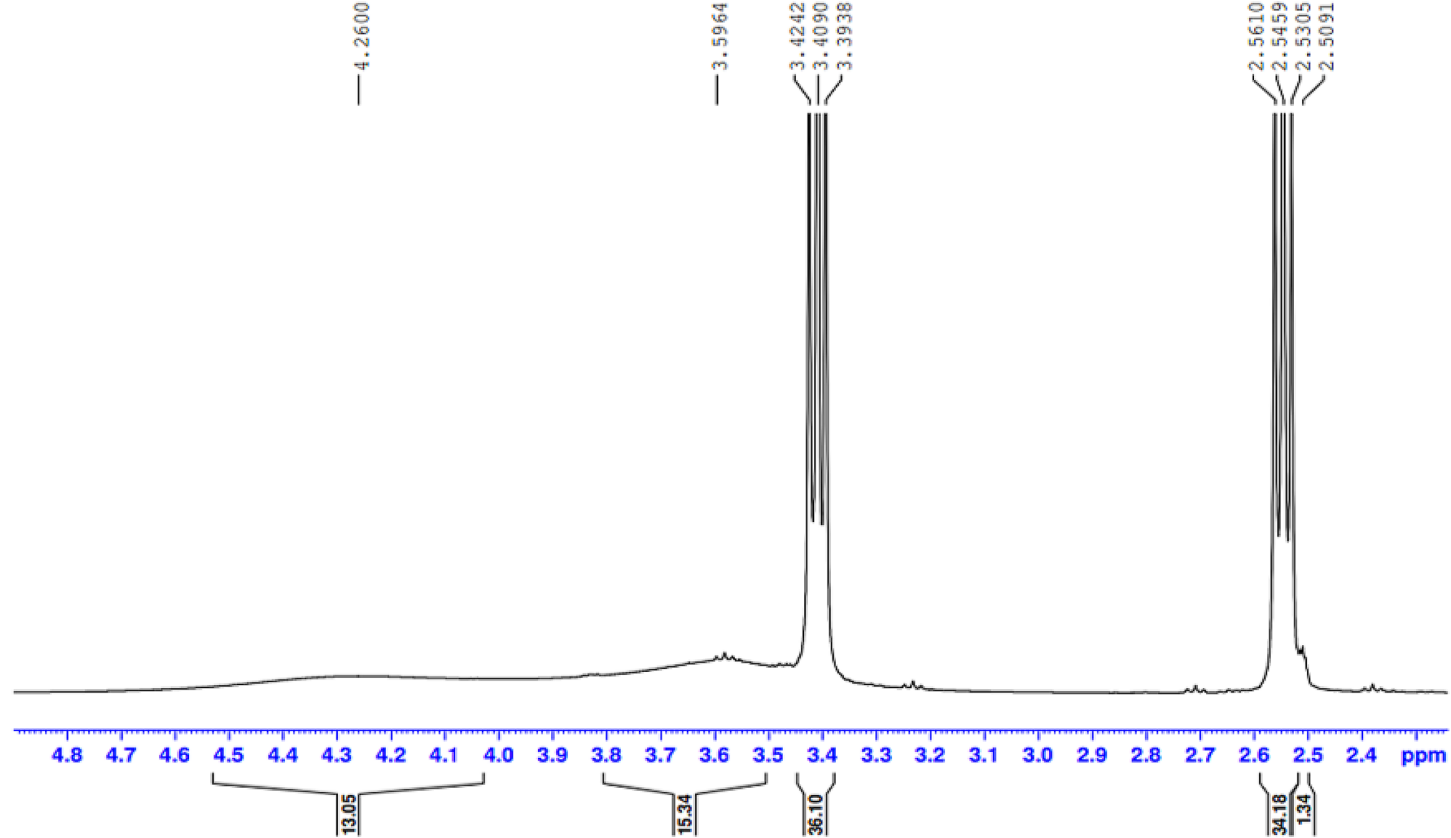
^1^HNMR spectrum of the prepared compound (B)

**Fig. 5:**
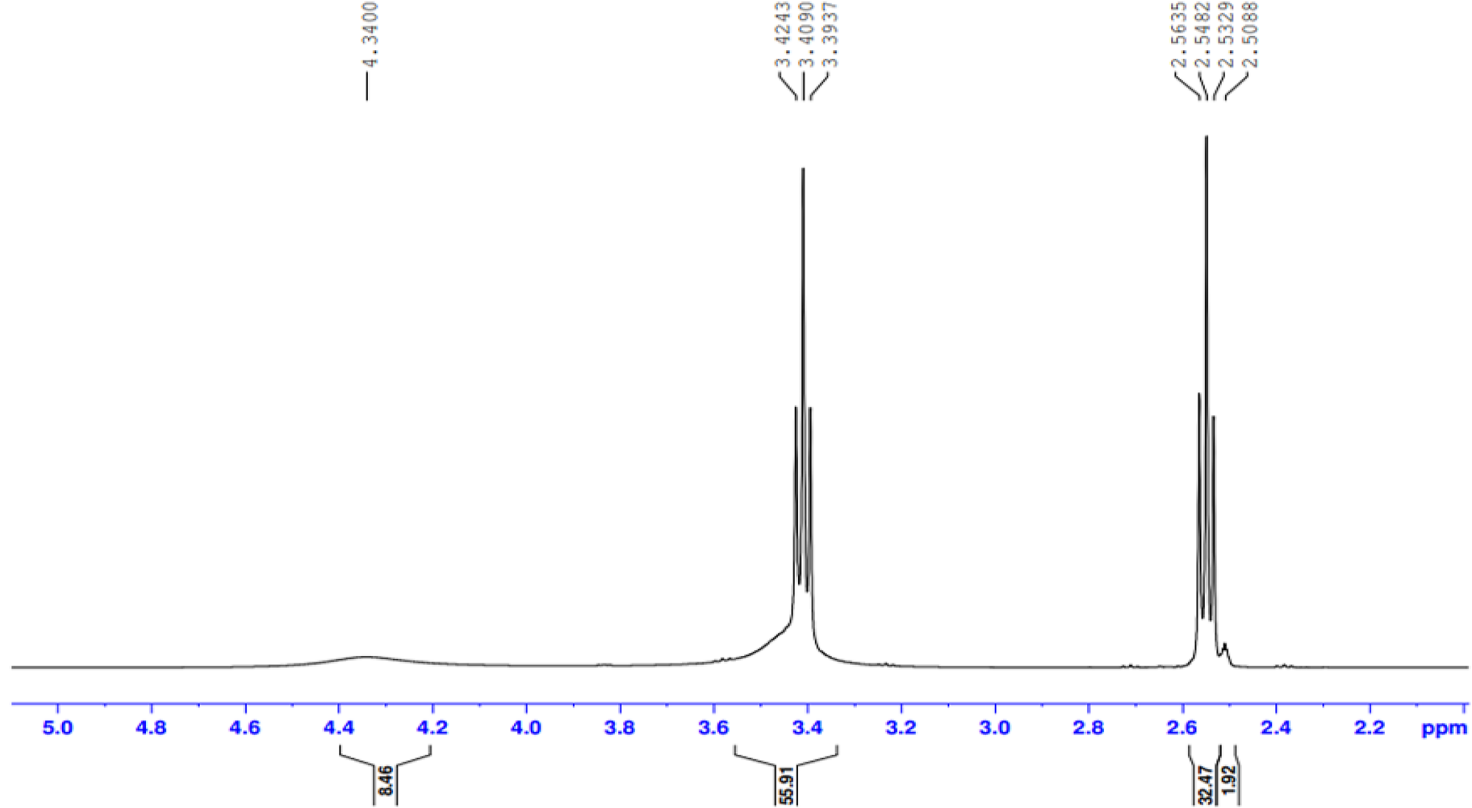
^1^HNMR spectrum of the prepared compound (C)

**Fig. 6:**
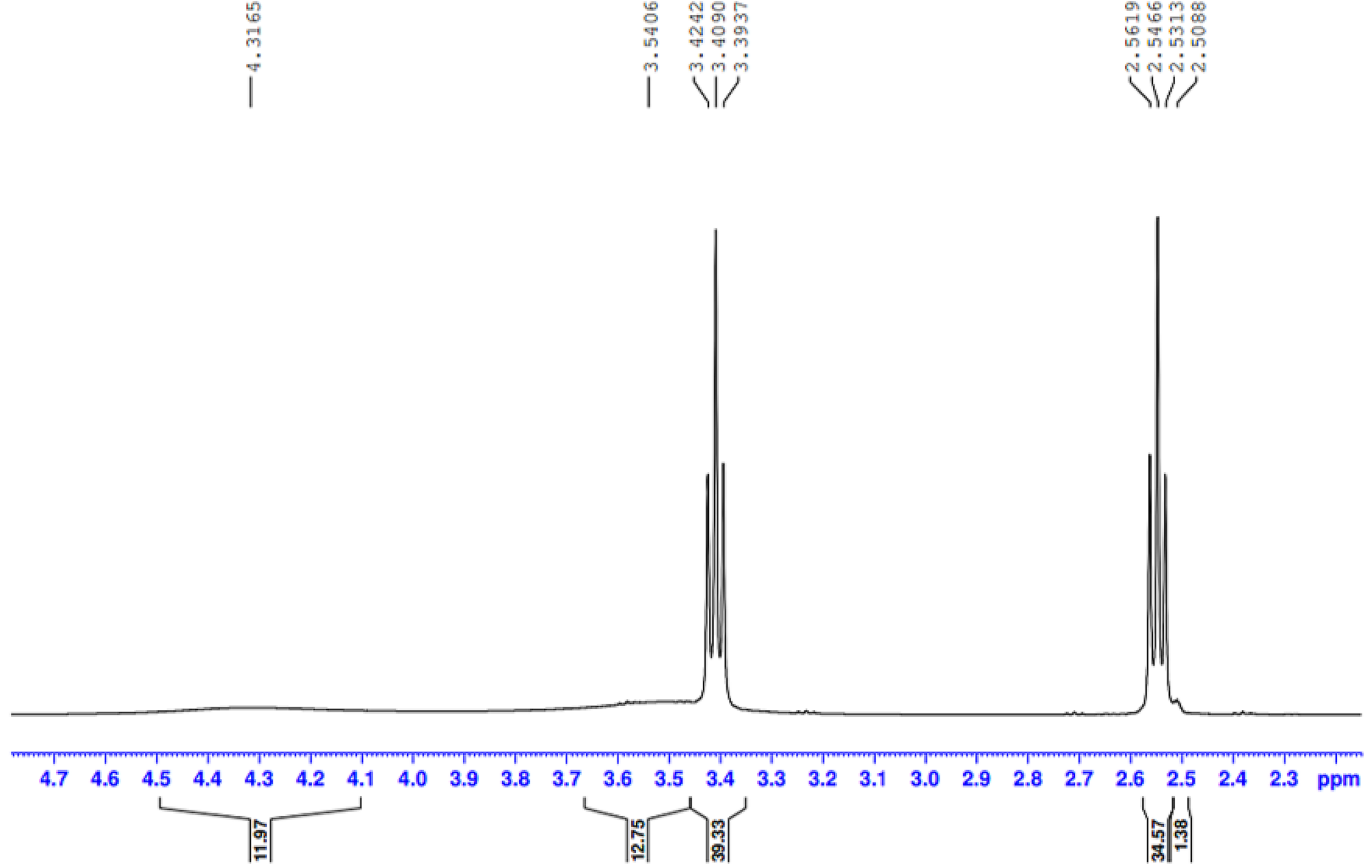
^1^HNMR spectrum of the prepared compound (D)

**Fig. 7:**
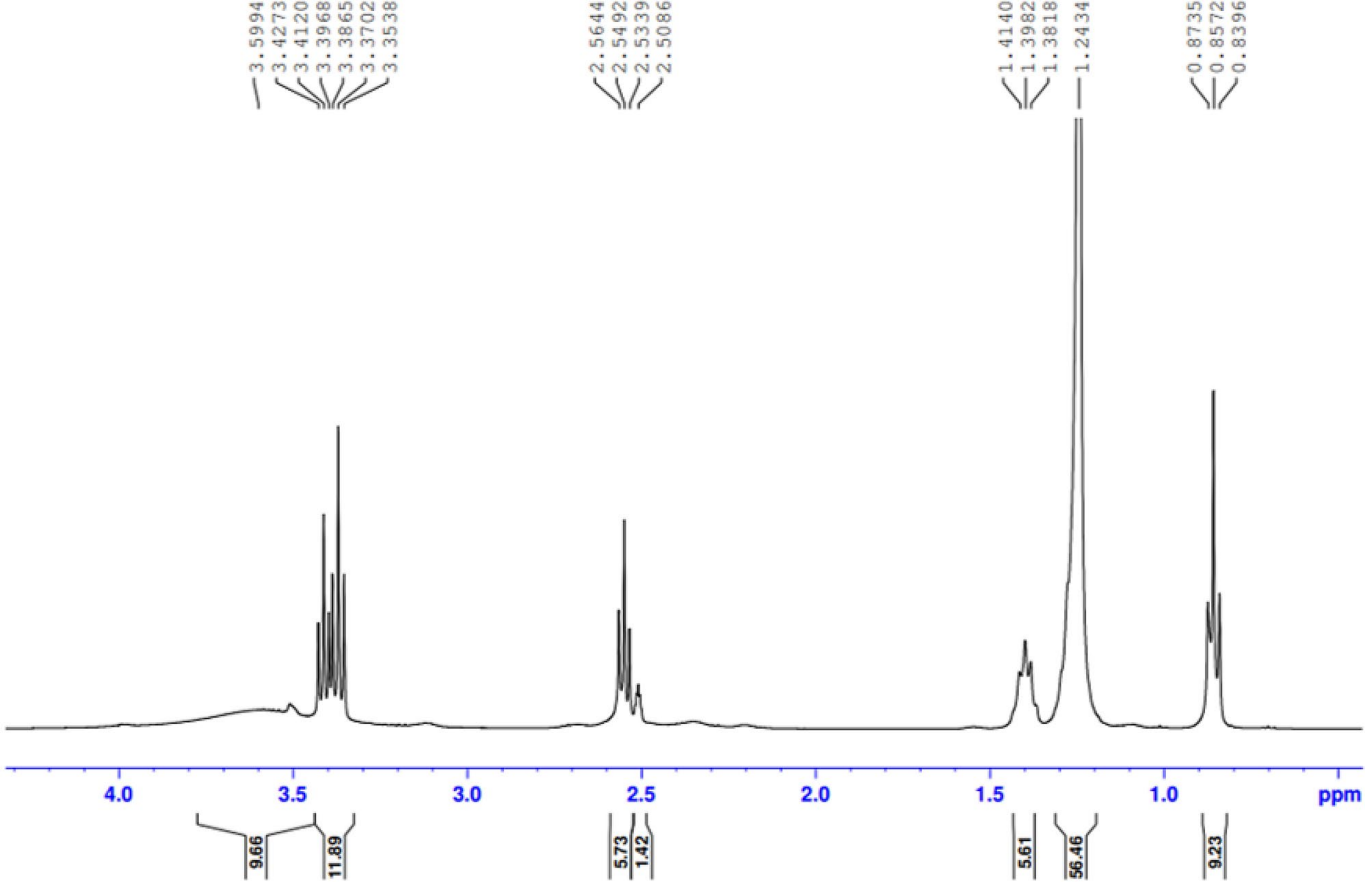
^1^HNMR spectrum of the prepared compound (E)

The presence of the ester group O = C-O-R band at 1732 cm-1 revealed that the ester termination of the synthesised E sample had formed. Moreover, Figs. (3, 4, 5, 6-7) show ^**1**^**HNMR chart** Protons of 5 hyperbranched polymers. Peaks of samples A to E have almost the same spectra that show: CH-NR protons at (1.3 − 1.5 ppm), NH_2_-O-C = O protons at (3.3-3.5 ppm) and R-NH protons at (2.3-2.6 ppm), the peak at about 2.5 ppm is certified to the remaining hydrogen protons of the solvent (DMSO). All the above peaks approve the construction of hyperbranched polymers.

### Dynamic light scattering (DLS)

**Fig. 8:**
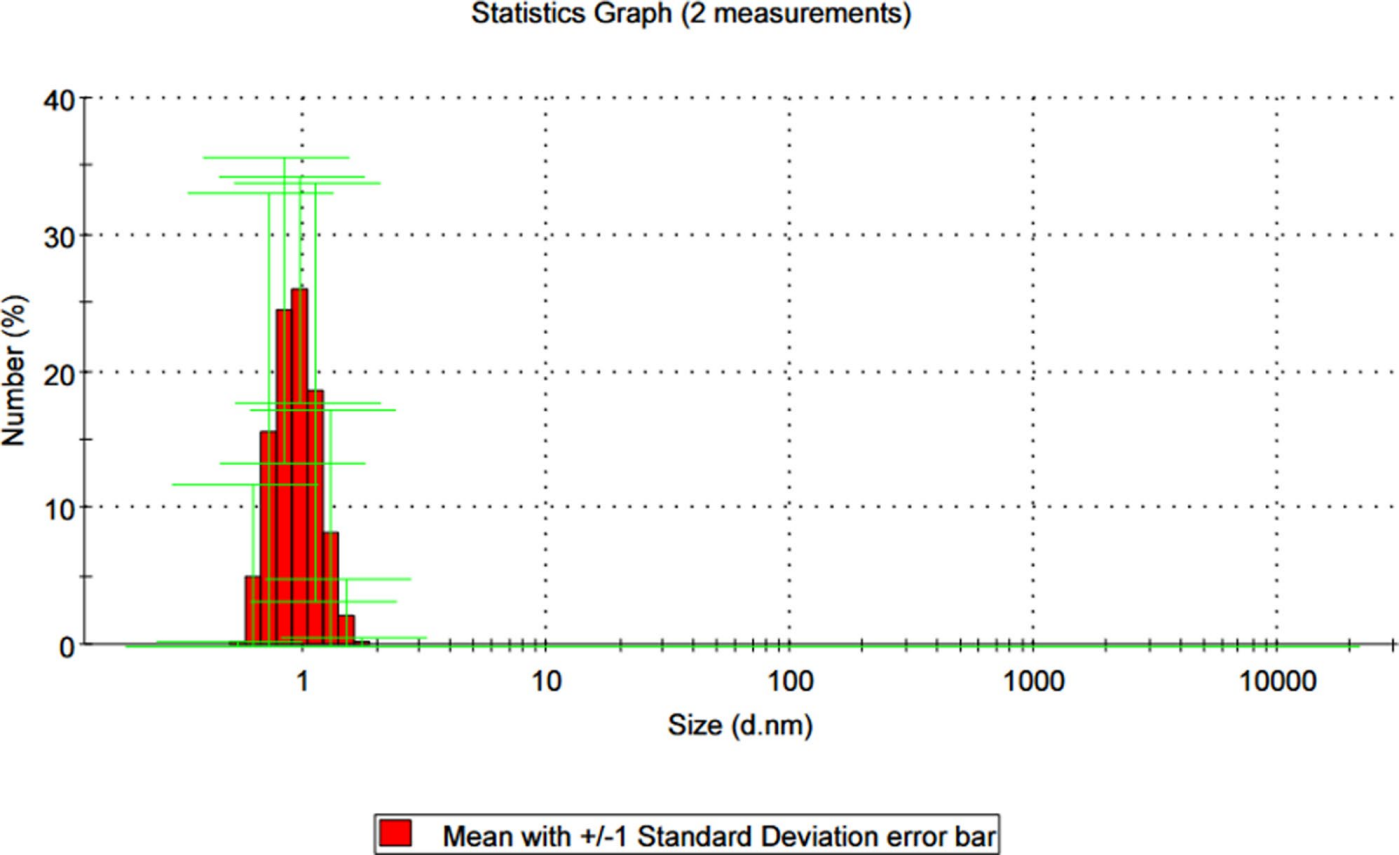
DLS image of prepared hyper branched polymers

DLS is another method commonly used to investigate the structure of macromolecules [25]. In this work, the particle size distribution of synthesised samples with various terminations was measured using the DLS. Figure (8) and Table (2).

**Table 2:** Molecular weights and particle sizes of prepared hyperbranched polymers

Sample	Molecular weight (g/mol)	Polydispersity	Diameter (nm)
**A**	8456	1.20	63
**B**	11,787	1.20	45
**C**	12,900	1.10	28
**D**	15,700	1.10	16
**E**	17,200	1.05	1.1

To protonate the terminal primary amine group, the samples were dissolved in 2% diluted distilled water, high dilution methanol, and 0.01% HCl. The data revealed that increasing the molecular weights and the number of branches increases particle size. This result could be attributed to the disruption of ordering molecules caused by increasing the branched moiety. It is clear from the data of particle size that increases, the particle size increases in the order of A˃B˃C˃D˃E. This means that, the size of prepared polymers increases by increasing terminal amine chains (TETA). This may be attributed to, as the terminated ended chains increase the regular structure (ordering structure) may be formed with large surface area and small particle size [25]. One possible explanation for this phenomenon is that as the number of terminated chains increases, the formation of a more regular or ordered structure occurs within the polymer matrix. This ordered structure may have a larger surface area and result in smaller particle sizes when dispersed in solution. In summary, the insights gained from DLS analysis suggest a complex interplay between molecular structure, branching, and terminal functional groups in hyperbranched polymer lube oil additives. Understanding these relationships can inform the design and optimization of polymer additives with tailored properties, ultimately enhancing their performance in lubricant applications.

### Differential scanning calorimetry (DSC)

**Fig. 9:**
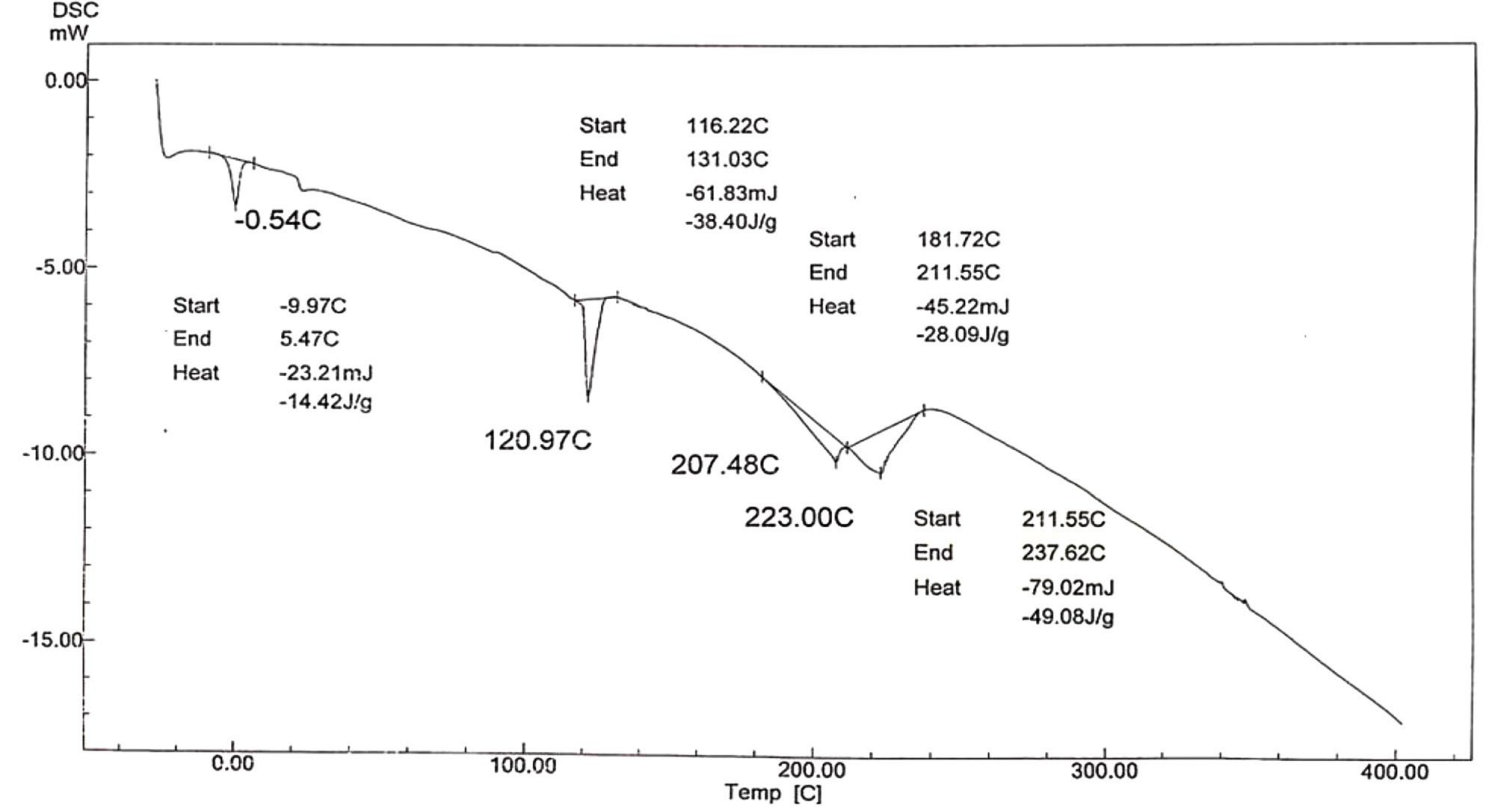
DSC Analysis of prepared hyper branched polymer (A)

**Fig. 10:**
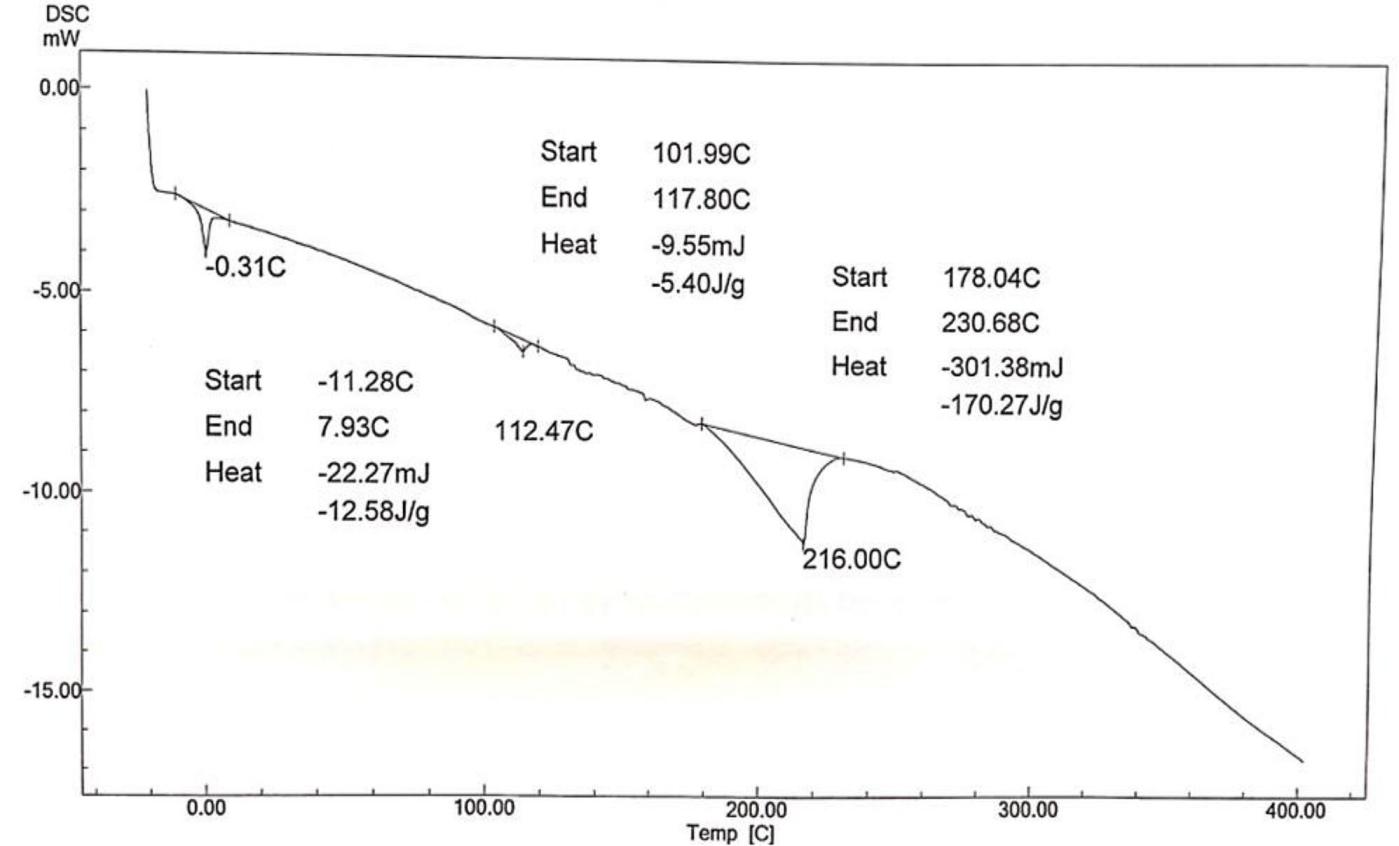
DSC Analysis of prepared hyper branched polymer (C)

**Fig. 11:**
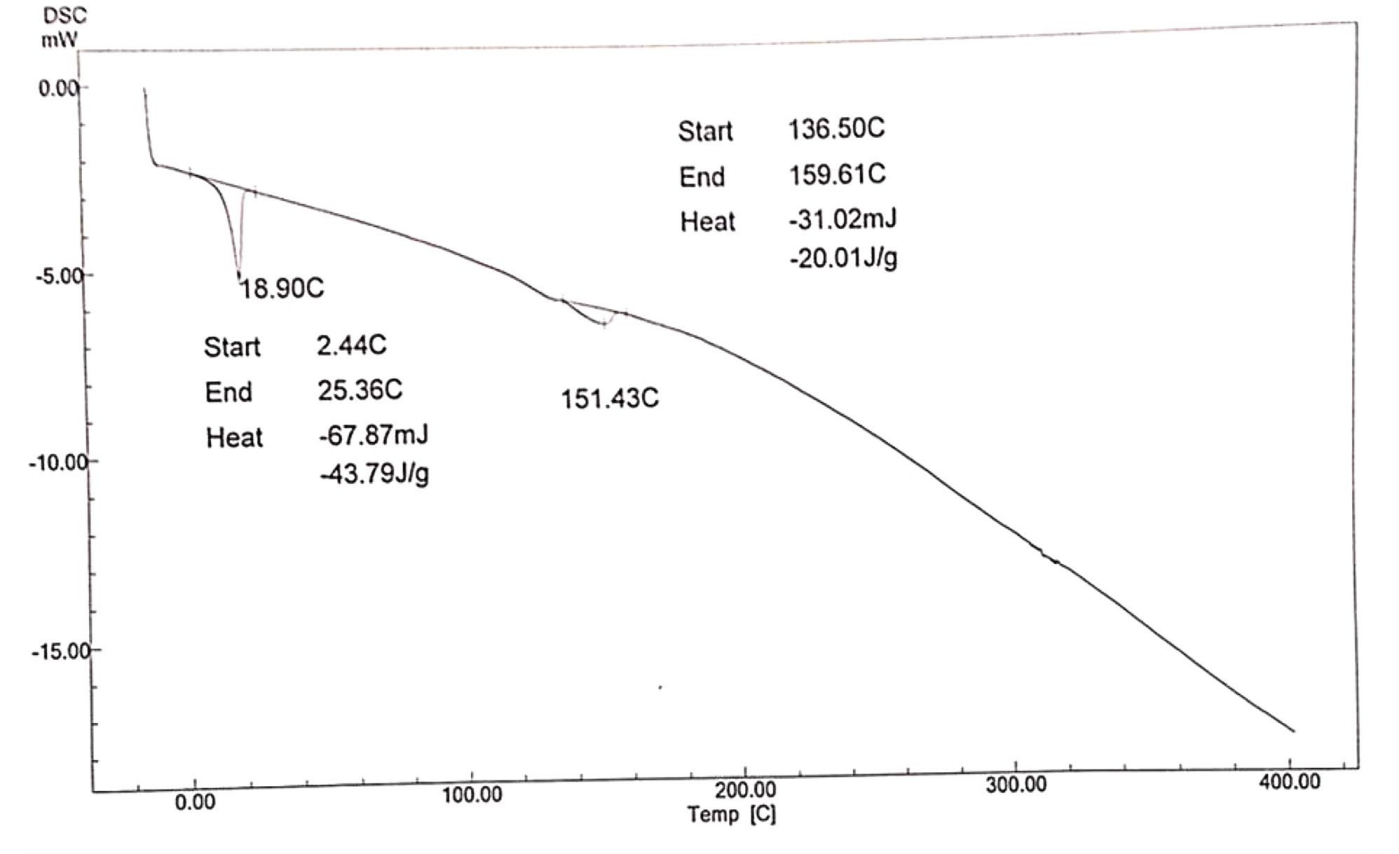
DSC Analysis of prepared hyper branched polymer (E)

DSC was applied to investigate the thermal behavior and classify the polymers into amorphous polymers and semi-crystalline by using cooling run and heating run which identify the glass transition temperature (*Tg*), melting temperature (*Tm*), and crystallisation temperature (*Tc*), and enthalpies, making DSC a useful tool for creating phase diagrams for various chemical systems. In this work Fig. (9). shows DSC Analysis of prepared hyper branched polymer (A) is endothermic peaks are observed, (*T*_m_) is 223 °C and the enthalpy − 49.08 J/g. Also Fig. (10). shows DSC Analysis of prepared hyper branched polymer (C) it is endothermic peaks are observed, (*T*_m_) is 216 °C and the enthalpy − 170.27 J/g. the different between the structure of A and C give increase in enthalpy due to increase the dodecyl acrylate ratio but Fig. (11). show DSC Analysis of prepared hyper branched polymer (E) it is endothermic peaks are observed, (*T*_m_) is 151.43 °C and the enthalpy − 43.79 J/g. The more negative the enthalpy of formation is for a substance, the greater its thermal stability so from the above result the more stable one is hyper branched polymer (C). All the prepared hyper branched polymer are (crystalline like structures this confirm that the prepared hyberpranched polymers have an ordinary shape and seems like dendrimers). Differential scanning calorimetry suggests that the prepared hyperbranched polymers resemble dendrimers in their shape and structure. Dendrimers are highly branched, tree-like molecules with well-defined structures and uniform molecular weights. While hyperbranched polymers may not possess the precise hierarchical structure of dendrimers, they share similarities in their branched architecture. The resemblance to dendrimers implies that the hyperbranched polymers exhibit a high degree of branching and complexity in their molecular structure. This structural intricacy can influence their thermal behavior and crystallinity, as the branching architecture may hinder molecular mobility and promote the formation of ordered regions. Overall, DSC analysis of hyperbranched polymer lube oil additives can confirm their crystalline-like structures and provide insights into their thermal properties. By understanding the relationship between molecular structure and thermal behavior, researchers can optimize the synthesis and formulation of these additives to enhance their performance in lubricant applications.

### Evaluation of prepared hyperbranched polymers as lube oil viscosity index improver

**Fig. 12:**
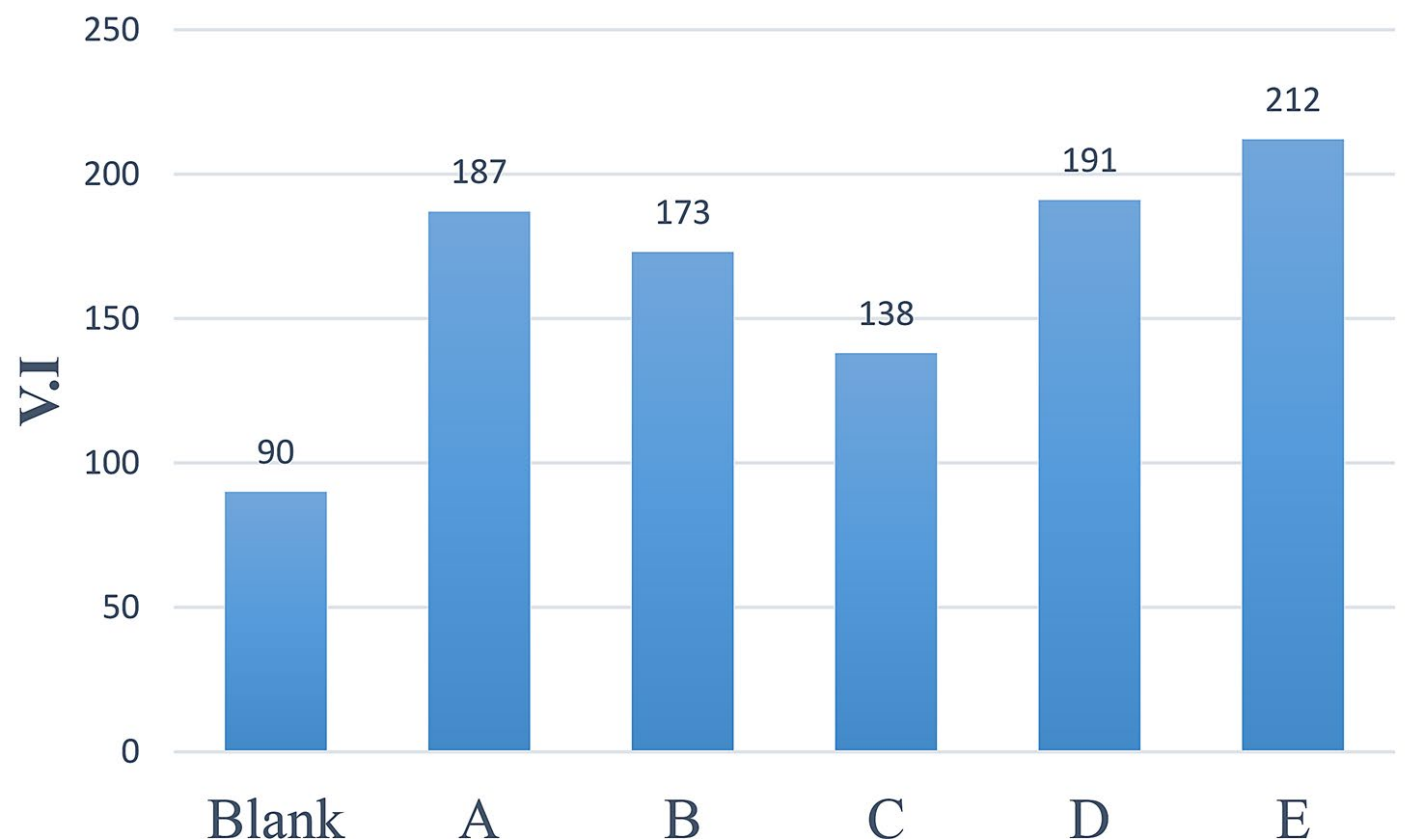
Viscosity index of prepared samples at 3%of additives concentration

The dispersion phase behavior of hyperbranched polymer molecules is critical to the polymeric Viscosity index enhancer's function (base oil). The efficiency of the soluble hyperbranched polymers as base oil viscosity index enhancers was assessed in accordance with ASTM D2270 (SAE 30) between 40 and 100 °C, for example, the kinematic viscosity of the oil that has not been doped and the oil that includes various amounts of the tested additives, was measured then calculated the viscosity index as shown in the Table (3) and Fig. (12) To examine the impact of the additive concentration, various additive concentrations ranging from 0 to **30 × 10**^**− 3**^**ppm** of the synthesized additives were employed. The VI rises when the prepared additives' concentration in the solution is increased. The lubricating oil viscosity reduces as the temperature rises, while the hyperbranched polymer molecule expands as a result of the rise in solvation power, and the micelle size rises. This drop in lubricating oil viscosity is balanced by the expansion of micelles, which reduces the fluctuations in viscosity with mixture temperature. As the concentration of the polymer increases, the overall volume of hyperbranched polymer micelles in the oil solution increases.

**Table 3:** Effect of concentration on the viscosity index

Conc. x10^-3^ ppm	A	B	C	D	E
30.00	187	173	138	191	212
20.00	183	169	130	185	209
10.00	175	154	121	181	202
5.00	172	140	110	176	198
2.50	169	121	106	169	192
0.00	90	90	90	90	90

The viscosity index of a high concentration polymer will thus be greater than that of a low concentration polymer. On the other hand, VI rises also when the percentage of triethylenetetramine as shown in prepared hyperbranched polymers (A, D and E ) although VI decreases by increasing the percentage of dodecyl acrylate s shown in prepared hyperbranched polymers (A, B and C) and the most efficiency one as VII is (E) VI = 212 which is higher than previous work [5, 7, 24, 29-31] because it has different functional group as mention before.

The described behavior of hyperbranched polymer lube oil additives suggests several unique characteristics that differentiate them from other types of additives:

1. Enhanced Viscosity Index Improvement: As the concentration of hyperbranched polymer additives increases in the lubricant solution, the viscosity index rises. This indicates that these additives have a significant impact on improving the viscosity-temperature relationship of the lubricant. Unlike some conventional additives whose effectiveness may plateau or diminish at higher concentrations, hyperbranched polymers continue to enhance the viscosity index even at elevated concentrations.
2. Temperature-Responsive Behavior: The lubricating oil viscosity typically decreases as temperature rises, which can lead to performance challenges in lubricant formulations, particularly at higher operating temperatures. However, hyperbranched polymer molecules exhibit expansion as a result of increased solvation power at higher temperatures. This expansion contributes to the enlargement of micelles formed by the polymer in the oil solution, which in turn helps to balance the drop in lubricating oil viscosity. This temperature-responsive behavior of hyperbranched polymers allows for better control and stabilization of viscosity over a wide temperature range.
3. Effective Micelle Formation: The expansion of hyperbranched polymer molecules leads to an increase in micelle size in the oil solution. These micelles act as carriers for the polymer additives, facilitating their dispersion and interaction with the lubricating oil. The larger micelle size resulting from higher polymer concentrations enhances the overall volume of hyperbranched polymer micelles in the solution. This increased volume contributes to improved lubricant performance by reducing viscosity fluctuations with changes in temperature.
4. Stabilization of Lubricant Properties: The ability of hyperbranched polymers to expand and form larger micelles in the oil solution helps stabilize the viscosity-temperature relationship. By counteracting the decrease in lubricating oil viscosity at elevated temperatures, hyperbranched polymer additives mitigate the adverse effects of temperature variations on lubricant performance. This stabilization effect contributes to smoother and more consistent lubricant behavior under changing operating conditions.

Overall, the unique combination of viscosity index improvement, temperature-responsive behavior, effective micelle formation, and stabilization of lubricant properties distinguishes hyperbranched polymer additives from other types of lubricant additives. These characteristics make hyperbranched polymers particularly well-suited for enhancing the performance and stability of lubricant formulations across a wide range of operating conditions.

### The rheological behavior

**Fig. 13:**
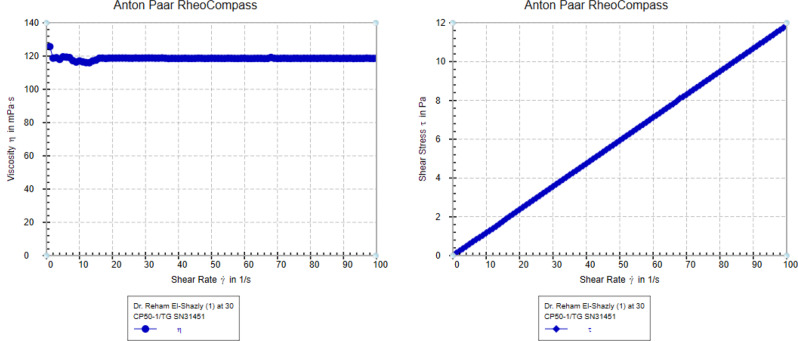
The rheological behavior of lubricating oil with additive concentration 3% (E)

For all the prepared hyperbranched polymers is the same for example (E) at concentration 30 × 10^− 3^ ppm the most efficiency one as VII which show in Fig. (13). The fluid has a Newtonian rheological behavior, which means that it meets the requirements with Newton's law of viscosity. Shear rate has no effect on viscosity [31-35]. 

For lubricant additives, the importance of a fluid exhibiting Newtonian rheological behavior, where shear rate has no effect on viscosity, lies in several key factors:

Consistent Lubricant Performance:

1. Consistent Lubricant Performance: In lubrication applications, maintaining a constant viscosity regardless of shear rate ensures consistent lubricant performance across a range of operating conditions. This predictability is crucial for ensuring that the lubricant provides reliable protection and reduces friction under varying loads and speeds.
2. Uniform Film Thickness: Newtonian behavior helps ensure that the lubricant film formed between moving surfaces remains uniform in thickness. This is essential for preventing metal-to-metal contact, minimizing wear, and providing effective boundary lubrication in machinery and equipment.
3. Stable Viscosity Index: Newtonian additives contribute to the stability of the lubricant's viscosity index. A constant viscosity ensures that the lubricant maintains its desired flow properties and lubricating effectiveness over a wide range of temperatures and operating conditions.
4. Simplified Formulation: Lubricant formulations containing Newtonian additives can be more straightforward to design and optimize. Since the viscosity of the lubricant remains constant, it simplifies the selection and blending of additives to achieve the desired performance characteristics without needing to compensate for variations in viscosity with shear rate.
5. Ease of Application: Lubricants with Newtonian additives are easier to apply and distribute uniformly over surfaces due to their predictable flow behavior. This facilitates the lubrication process and ensures that the lubricant reaches critical contact points effectively.
6. Compatibility with Equipment: Newtonian lubricant additives are compatible with a wide range of machinery and equipment, as they provide consistent lubrication performance without causing adverse effects on system components.

Overall, the important effect of lubricant additives exhibiting Newtonian rheological behavior is their ability to maintain a constant viscosity regardless of shear rate. This ensures consistent lubricant performance, stable viscosity index, simplified formulation, and ease of application, ultimately contributing to improved machinery efficiency, reliability, and longevity.

## Conclusion

To improve the synthesis's reliability, the prepared branched polymers with different nano-sized terminates (A to E) were successfully synthesised using an easy and commercialised method according to repeated Michale addition and amidation. The particle size of the synthesised samples grows as the molecular weights and number of branching increase.

The greater the thermal stability of a substance, the greater its negative enthalpy of formation; thus, hyper branched polymers are more stable (C). All the hyperbranched polymers that have been prepared are (crystalline).

The use of branched polymers with varied terminations as viscosity index enhancers has proved effective. As the concentration of prepared additives in the solution increases, so does the VI., as does the percentage of triethylenetetramine in prepared hyperbranched polymers, but it decreases as the percentage of dodecyl acrylate in prepared hyperbranched polymers increases, and the most efficient one as VII is (E) VI = 212, Newtonian rheological behavior is observed in all the prepared hyperbranched polymers.

## Data Availability

The data that support the findings of this study are available from the corresponding author upon reasonable request.
